# Cost-effectiveness analysis of the tislelizumab versus docetaxel for advanced or metastatic non-small-cell lung cancer in China

**DOI:** 10.3389/fpubh.2024.1425734

**Published:** 2024-07-18

**Authors:** Xiaoyu Zhang, Xiongxiong Fan, Jin Zhang, Fengli Jiang, Yiping Wu, Beibei Yang, Xinghuan Li, Dong Liu

**Affiliations:** ^1^Clinical Pharmacy Office, Baoji Central Hospital, Baoji, Shaanxi, China; ^2^Department of Pharmacy, Xi’an Jiaotong University Health Science Center, Xi’an, Shaanxi, China

**Keywords:** NSCLC, tislelizumab, docetaxel, Markov model, cost-effectiveness

## Abstract

**Background:**

Tislelizumab is the first PD-1 inhibitor in China to demonstrate superior efficacy in second-line or third-line treatment of patients with advanced or metastatic non-small-cell lung cancer (NSCLC). This study aimed to evaluate the cost-effectiveness of tislelizumab compared to docetaxel from a Chinese healthcare system perspective.

**Methods:**

A dynamic Markov model was developed to evaluate the cost-effectiveness of tislelizumab in comparison to docetaxel in second or third-line treatment. The efficacy data utilized in the model were derived from the RATIONALE-303 clinical trial, while cost and utility values were obtained from the drug data service platform and published studies. The primary outcomes of the model encompassed quality-adjusted life years (QALYs), costs, and incremental cost-effectiveness ratios (ICERs). One-way sensitivity analysis and probabilistic sensitivity analysis were conducted to validate the robustness of the base case analysis results.

**Results:**

The tislelizumab group demonstrated a cost increase of CNY 117,473 and a gain of 0.58 QALYs compared to the docetaxel group, resulting in an ICER value of CNY 202,927 per QALY gained.

**Conclusion:**

The administration of tislelizumab in patients with advanced or metastatic NSCLC not only extends the progression-free survival (PFS) and overall survival (OS). Moreover, this treatment demonstrates a favorable cost-effectiveness profile across the Chinese population.

## Introduction

In 2022, China witnessed a total of 870,000 incident cases and 770,000 fatalities attributed to lung cancer,which is the most prevalent and lethal malignant tumor in our country (1). NSCLC constitutes approximately 80–85% of all lung cancer cases and demonstrates a mere 15% five-year survival rate (2, 3). Due to the inconspicuous onset of early NSCLC, a considerable proportion of patients failed to capitalize on the optimal treatment window (4, 5).

Platinum-based chemotherapy has traditionally been the standard first-line treatment for patients with driver-negative advanced or metastatic NSCLC, while PD-1/PD-L1 inhibitors have demonstrated comparable efficacy and improved tolerability as second-line treatment following disease progression after initial chemotherapy (6–8). Regarding options for second-line or third-line treatment in these patients, the NCCN Guidelines (2023) (9) recommend PD-1/PD-L1 inhibitors (Category 2A) such as nivolumab, pembrolizumab, and atezolizumab, or chemotherapeutic agents like docetaxel, pemetrexed, and gemcitabine (Category 2B). However, previous studies (10–14) have demonstrated that economic evaluations of imported PD-1/PD-L1 inhibitors, such as pembrolizumab, nivolumab, and atezolizumab, do not confer a cost-effectiveness advantage. As a domestically developed PD-1/PD-L1 inhibitor, tislelizumab offers a lower price compared to imported counterparts while maintaining comparable efficacy (12). Consequently, the CSCO guidelines advocate for tislelizumab as second-line treatment in patients with driver-negative advanced or metastatic NSCLC. A study RATIONALE 303 (15) evaluating tislelizumab, a domestically developed PD-1 inhibitor, demonstrated significant improvements in progression-free survival (PFS) and overall survival (OS) compared to docetaxel among patients with advanced NSCLC. In the final data analysis, compared with docetaxel, tislelizumab’s OS (median 19.3 versus 11.5 month, respectively; HR = 0.53, *p* < 0.0001) and PFS (median 4.2 versus 2.6 month, respectively; HR = 0.63, *p* < 0.0001) were both statistically and clinically significant.

Despite cost-effectiveness analyses comparing tislelizumab and docetaxel have been conducted (16, 17), these studies possess certain limitations. Firstly, it should be noted that the inclusion of tislelizumab in China’s national healthcare security special drug management scope in 2021, along with subsequent price reductions in March 2023 and after the centralized purchase of docetaxel, has resulted in a disparity between the treatment costs of these two drugs compared to those considered in previous studies. Additionally, the published cost-effectiveness analysis is based on interim data from the RATIONALE 303 study (18). However, it is important to acknowledge that final data reveals sustained improvement in OS within the tislelizumab group when compared to the docetaxel group, thereby indicating enhanced clinical efficacy. All aforementioned factors may potentially impact the cost-effectiveness evaluation of tislelizumab versus docetaxel.

Based on the final clinical efficacy data obtained from the RATIONALE 303 study (15), and considering the prevailing prices of tislelizumab and docetaxel in China, this study establishes a model and formulates an evaluation plan to assess the cost-effectiveness of tislelizumab compared to docetaxel for the treatment of advanced or metastatic NSCLC from a health service system perspective. This analysis offers valuable insights for clinical treatment decision-making, dynamic adjustments to national healthcare coverage lists, and drug price negotiations.

## Materials and methods

### Patients and intervention

The present study was grounded in a phase 3 clinical trial (RATIONALE-303) that included patients (≥18 years of age) diagnosed with histologically confirmed locally advanced or metastatic squamous or non-squamous NSCLC. These patients had previously undergone one or two treatments, which included platinum-based dual chemotherapy. Individuals who had been treated previously with immune checkpoint inhibitors (ICIs), as well as those carrying EGFR mutations or ALK gene translocations, were excluded from this study. A total of 805 patients were randomized 2:1 to tislelizumab and docetaxel.

In the RATIONALE-303 trial, as a second-line or third-line treatment strategy, two treatment groups were assigned: tislelizumab 200 mg administered every 3 weeks or docetaxel 75 mg/m^2^ given every 3 weeks until disease progression according to Response Evaluation Criteria in Solid Tumors (RECIST) version 1.1, intolerable toxicity, or withdrawal of consent.

### Model structure

A Markov model was employed in this study to establish three mutually exclusive cycle states, namely PFS, progressive disease (PD), and death, based on the outcome of NSCLC disease course and clinical trial data. All patients were initially enrolled in the PFS state. Depending on the probability of metastasis, some patients remained in the PFS state while others transitioned to the PD or death state after each cycle. The patients transitioning to the PD state varied with each cycle; some remained in PD while others progressed to death. Eventually, all patients reached a terminal state of death after several cycles. The model utilized a 3-week cycle duration over a research period of 12 years, totaling 208 cycles. Dynamic transfer probabilities were incorporated to capture disease progression risk over time better.

### Analyzing clinical data

The OS and PFS curves in RATIONALE-303 (15) were derived through point selection utilizing the web-based software WebPlotDigitizer (Version 4.6).^1^ Following the approach proposed by Guyot et al. (19), we reconstructed and extrapolated the Kaplan–Meier curve using R 4.2.2. The reconstruction curves and relevant statistical tests are presented in Supplementary Figures S1, S2, and Supplementary Table S1, demonstrating a favorable reconstruction outcome. To fit the individual-level data, we employed exponential, gamma, generalized gamma, Gompertz, Weibull, log-logistic, and log-lognormal distributions. According to Akaike information criterion (AIC) and Bayesian information criterion (BIC) results (Supplementary Table S2), the log-logistic distribution was employed in this study to fit the survival curve. The scale parameter α and shape parameter β of the function were obtained as presented in Table 1. Subsequently, utilizing the log-logistic distribution function S(x) = 1-F(x,α,β)= $1-\frac{1}{1+{\left(\frac{x}{\alpha }\right)}^{-\beta }}$ where S represents the survival rate and x denotes time, we determined the transition probability between states.

**Table 1:** Base-case key model inputs.

Parameter	Values	Range	Distribution	Sources
Survival model for tislelizumab				
Log-logistic model for OS	Scale (α)=17.43252; Shape (β)=1.48925		-	(15)
Log-logistic model for PFS	Scale (α)=4.84787; Shape (β)=1.42274		-	(15)
Survival model for docetaxel				
Log-logistic model for OS	Scale (α)=11.7162; Shape (β)=1.5699		-	(15)
Log-logistic model for PFS	Scale (α)=3.11381; Shape (β)=2.12956		-	(15)
Costs (CNY)				
Tislelizumab per cycle	2675	2140~2675	Gamma	Local charge
Docetaxel per cycle	419.25	419.25~898.36	Gamma	Local charge
Best supportive care per cycle	2467	1973.65~2960.47	Gamma	(20)
Severe AEs	2534	2027.2~3040.8	Gamma	(21)
Utilities				
PFS	0.804	0.724~0.884	Beta	(22)
PD	0.321	0.289~0.353	Beta	(22)
Neutropenia	-0.200	-0.220~-0.180	Beta	(22)
Febrile neutropenia	-0.42	-0.462~-0.378	Beta	(22)
Anemia	-0.078	-0.086~-0.070	Beta	(22)
Asthenia	-0.078	-0.086~-0.070	Beta	(22)
Other				
Discount rate	0.05	0~0.08	Fixed in PSA	(23)

### Cost inputs

The cost calculation in this study solely considered direct healthcare costs, as it was grounded within the context of the Chinese healthcare system. Direct medical costs encompass drug costs, adverse events (AEs) treatment costs, and best supportive care (BSC) costs. It was assumed that the cost of BSC includes doctors’ diagnosis and treatment expenses, material expenses, hospital bed expenses, nursing expenses, etc. For this study, the prices of tislelizumab and docetaxel were sourced from a public database.^2^ As tislelizumab is a national negotiated drug, its pricing remains relatively consistent nationwide, with the current price observed at CNY 1377.5/100 mg. Following the fifth round of national centralized procurement negotiations in 2021, the price of docetaxel has decreased and currently ranges between CNY 65 ~ 139.28/20 mg. To facilitate drug dose calculations, a mean body surface area (BSA) of 1.72 m^2^ and a body weight of 65 kg were assumed. Table 1 presents the medical costs for each cycle. The currency utilized in this study was RMB.

### Utility inputs

The present study is based on the recently published health utility values of NSCLC, which amalgamates health utility values from diverse countries. Specifically, data pertaining to the Chinese population has been selected for analysis in this investigation (22). The utility values for PFS and PD states were determined as 0.804 and 0.321, respectively. Furthermore, considering the impact of AEs on utility values, we incorporated grade ≥ 3 AEs with a frequency ≥ 5% observed in the RATIONALE-303 trial into our analysis. The disutility values assigned to neutropenia, febrile neutropenia, anemia, and asthenia were established as 0.2, 0.42, 0.078, and 0.078, respectively. To account for differences between tislelizumab and docetaxel groups regarding incidence rates of PFS and PD states, their respective utility values were converted accordingly.

### Base-case analysis

The Markov model was constructed using TreeAge Pro software, and the model outcomes included QALYs, total costs, and ICER. According to the China Guidelines for Pharmacoeconomic Evaluations (24), it is recommended that the threshold range for willingness to pay (WTP) should be 1 ~ 3 times the *per capita* GDP. In this study, we established the WTP threshold at 3 times the Chinese *per capita* GDP in 2022, with a discount rate of 5% (24).

### Sensitivity analysis

The impact of parameter variations (e.g., costs, utility, and discount rate) on the results was assessed through a one-way sensitivity analysis in this study. Regarding drug pricing, considering tislelizumab’s exclusive production, the subsequent price after national negotiation typically remains stable. Therefore, we establish the current price as the upper limit and reduce it by 20% to set the lower limit. The upper and lower limits for docetaxel prices are determined based on the highest and lowest bid prices in each region of the country. The utility parameter is allowed to vary within ±10% of its baseline value. A discount rate of 5% is applied with upper and lower limits ranging from 0 to 8%. To address uncertainty in model inputs, probabilistic sensitivity analysis (PSA) employing second-order Monte Carlo simulation with 5,000 iterations was conducted. The results were presented through cost-effectiveness scatter plots and cost-effectiveness acceptability (CEA) curves. Health utility values followed a beta distribution while costs followed a gamma distribution.

## Results

### Base-case analysis

The cost of the docetaxel group was CNY 69,111 for an incremental gain of 0.46 QALYs, whereas the tislelizumab group incurred a cost of CNY 186,583 to achieve an incremental QALY gain of 1.04. Compared to the docetaxel group, the tislelizumab group had a higher expenditure of CNY 117,473 but gained an additional 0.58 QALYs. The ICER was calculated as CNY 202,927 per QALY gained (Table 2), which falls below the WTP (CNY 257,016). These findings suggest that tislelizumab demonstrates superior cost-effectiveness and economic viability compared to docetaxel.

**Table 2:** Results of cost-effectiveness analysis.

Treatment	Total cost (CNY)	QALYs	Δcost (CNY)	ΔQALYs	ICER
Tislelizumab	186583	1.04	117473	0.58	202927
Docetaxel	69111	0.46	-	-	-

### Sensitivity analyses

**Figure 1:**
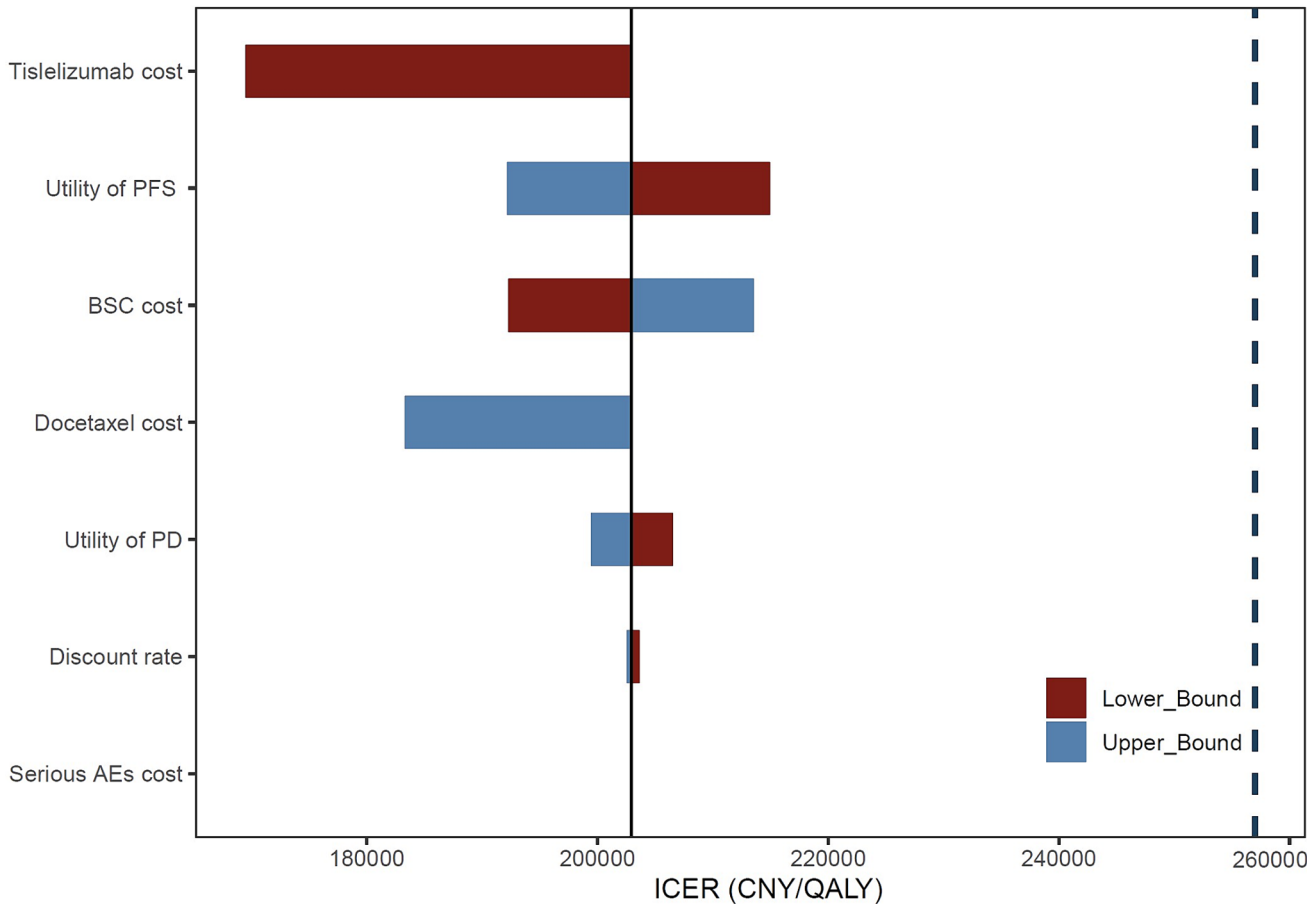
Tornado diagram of one-way sensitivity analysis. BSC, best supportive care; AEs, adverse events.

The one-way analysis results demonstrate (Figure 1) that the price of tislelizumab has the most significant impact on the ICER value of the entire model, followed by the utility value of PFS, the cost associated with BSC, and docetaxel price. Nevertheless, it is worth noting that regardless of variations in these parameters, the ICER value consistently remains below the WTP threshold, indicating a high level of stability within our model.

**Figure 2:**
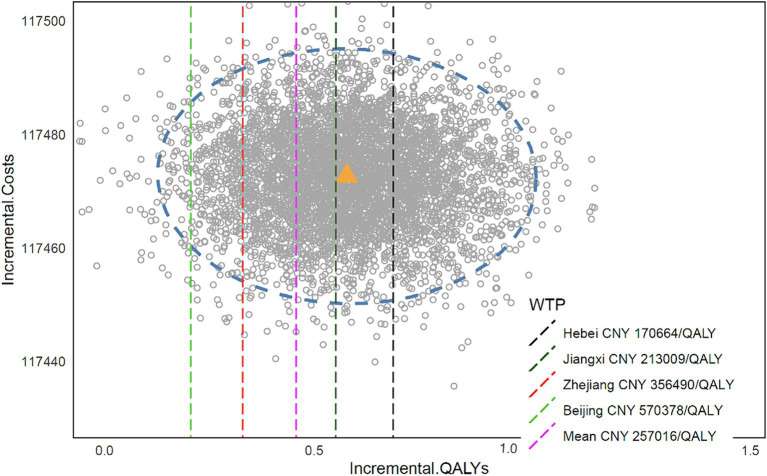
Monte Carlo scatter plot.

The results of the PSA analysis are presented in Figure 2, where the yellow dots on the vertical and horizontal axes represent the average cost and average QALY, respectively. About 74.8% of the dispersal points were located to the right of the average WTP threshold in China, indicating a 74.8% probability of tislelizumab having a cost-effectiveness advantage in China. In addition, we also counted the WTP thresholds for Chinese provinces, and we found that the probability of tislelizumab having a cost-effectiveness advantage based on the Beijing threshold was 98.0%, Zhejiang 91.4%, Jiangxi 55.9%, and Hebei 34.74%, with the remaining provinces not listed. According to statistics, a total of 21 provinces and cities in China have WTP thresholds greater than ICER.

**Figure 3:**
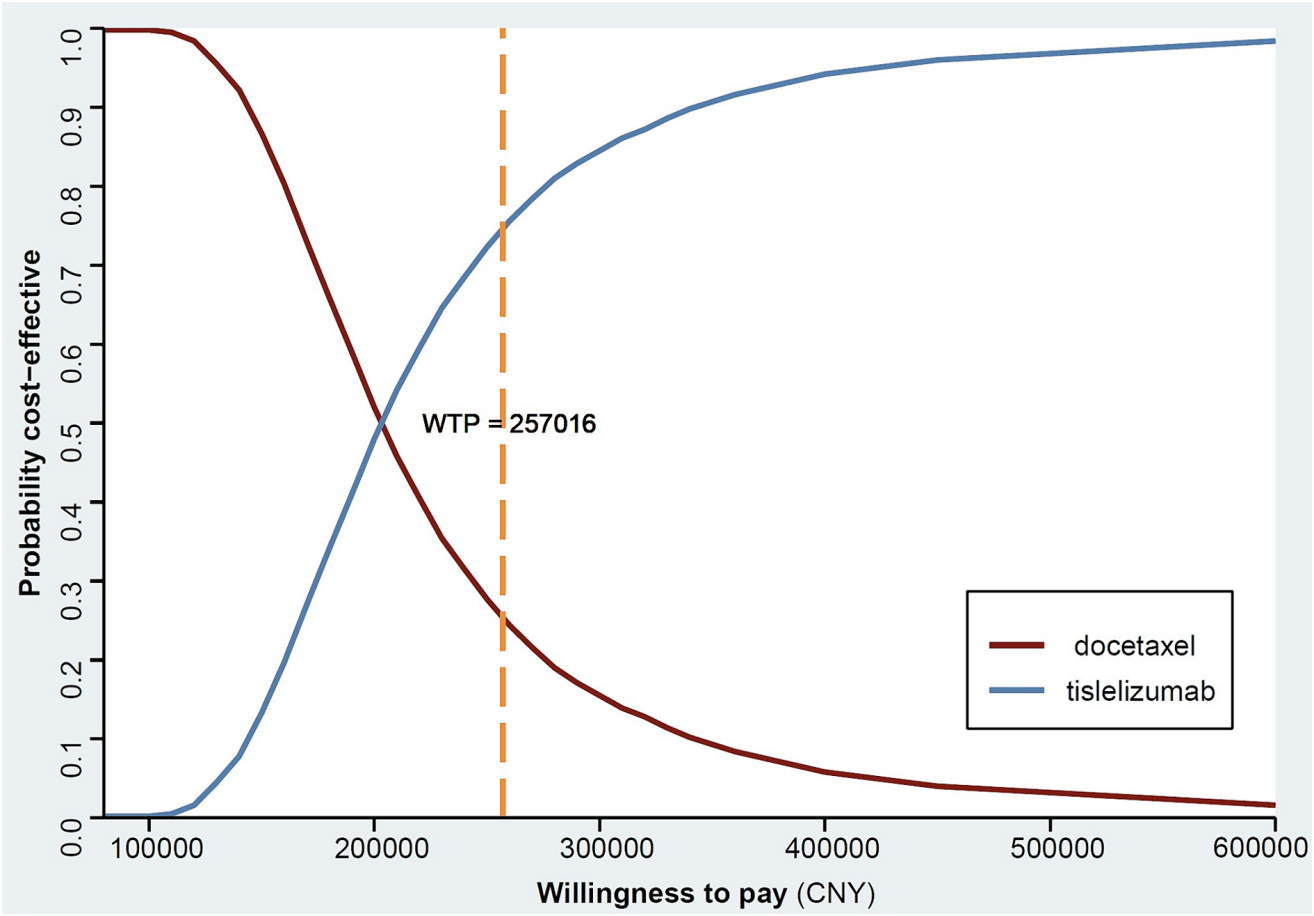
Cost effectiveness acceptability curve. WTP, willingness to pay.

The CEA curve (Figure 3) illustrates that at a WTP threshold below CNY 100,000, there is only a marginal probability (0.16%) of tislelizumab being cost-effective. However, when considering a higher WTP threshold such as CNY 500,000, approximately 97.0% of patients receiving tislelizumab demonstrate greater cost-effectiveness compared to those treated with docetaxel. Notably, as the WTP threshold increases further, there is an increasing likelihood that tislelizumab outperforms docetaxel in terms of cost-effectiveness.

## Discussion

This study substantiated the cost-effectiveness advantage of tislelizumab over docetaxel as a second-line or third-line treatment for patients with driver-negative advanced or metastatic NSCLC. It differed from previous published economic studies (16, 17) on tislelizumab and docetaxel in two aspects: (1) The final data was utilized in this study (15). Firstly, the final data demonstrated a significant enhancement in OS for the tislelizumab group compared to the docetaxel group, indicating a notable disparity in clinical efficacy. Secondly, employing the final data enables more precise fitting and extrapolation of Kaplan–Meier curves, thereby offering advantages in inferring long-term survival. (2) The latest drug prices were considered in this study. Tislelizumab was included in the healthcare security catalog on March 1, 2021, and after participating in medical insurance negotiations, its price underwent a reduction from CNY 10,688/100 mg to CNY 2,180/100 mg as reported in previous studies (16, 17). However, subsequent ongoing negotiations have led to a continuous decline in the price of tislelizumab; currently priced at CNY 1377.5 which represents an approximate reduction of 37%. Additionally, the price of docetaxel witnessed a decline subsequent to negotiations during the fifth batch of national centralized procurement conducted in 2021. In summary, this study comprehensively considered how changes to both tislelizumab and docetaxel’s latest prices impact treatment costs.

The results of the cost-effectiveness analysis in this study demonstrated that the tislelizumab group incurred an additional expenditure of CNY 117,473 while gaining an extra 0.58 QALYs. Previous studies, such as Gong et al. (16) reported that tislelizumab yielded an incremental gain of 0.51 QALYs at a supplementary cost of CNY 62,008. Furthermore, Zhou et al. (17) found that tislelizumab resulted in an additional increase of 0.33 QALYs with an incremental cost of CNY 62,459 (average exchange rate for 2022: $1 = CNY 6.7261). The findings from this study suggested that compared to previous research, tislelizumab can offer greater QALY gains at a relatively higher cost, but ICER is still economical when it is lower than WTP. In addition, according to the WHO criterion that the WTP threshold is 3 times GDP *per capita*, this study also calculates the WTP threshold of Chinese provinces in 2022, and finds that there are 21 provinces and cities in China where tislelizumab has a cost-effectiveness advantage. In other 11 provinces (Hainan, Henan, Yunnan, Qinghai, Xizang, Hebei, Jilin, Guizhou, Guangxi, Heilongjiang, Gansu, etc.), the WTP threshold was lower than ICER. It is believed that with the development of social economy and the improvement of *per capita* GDP, there will be more and more regions with cost-effectiveness advantages.

It should be noted that the value attributed to a patient’s time near end-of-life differs from their valuation during other stages of life cycle (25). In a study conducted by Yin et al. (26) on Chinese patients with NSCLC, it was observed that end-of-life patients placed greater importance on gains related to life expectancy. For instance, they were willing to pay $43,160 (equivalent to CNY 278,383 in 2021 USD) for each additional QALY in PFS. Notably, this willingness to pay exceeds China’s *per capita* GDP level of 242,928 yuan in 2021 by threefold. The National Institute for Health and Care Excellence (NICE) in the UK considers treatments with potential life expectancy extension exceeding 3 months for patients with less than 2 years’ prognosis under a higher WTP threshold (27). In other words, individuals nearing end-of-life may be more inclined to allocate additional resources in exchange for prolonged survival. Consequently, enhancing the average societal WTP would be advantageous for regions in China characterized by lower *per capita* GDP and limited economic development.

In terms of drug prices, tislelizumab is a national negotiated drug, and its price is basically uniform throughout the country; the price of docetaxel is based on the price of drugs purchased centrally by the state. The state’s centralized procurement is aimed at lowering drug prices. Since the drugs participating in the bidding must pass the generic drug consistency evaluation, the efficacy and safety of the drugs purchased centrally are comparable to that of the original drugs. To encourage hospitals to use centrally purchased low-cost drugs, the National Healthcare Security Administration (NHSA) has raised reimbursement standards to encourage patients to choose drugs in the centrally purchased list. In addition, the Local Healthcare Security Administration has the power to allocate the budget of public health facilities generated by health insurance, which ensures that hospitals receive the same level of funding even if they choose to use cost-effective winning drugs, and the funds saved by hospitals from the use of centrally procured drugs are mainly used to redistribute the salaries of hospital staff. These policies encourage doctors to prescribe centrally procured drugs. Therefore, centralized procurement of drugs is widely used in medical institutions (28–30). However, despite the incentives that have been put in place for physicians, providers, and patients, some physicians and patients may still use pricier original drugs, which, for this study, would have contributed to the cost-effectiveness advantage of tislelizumab if the pricier docetaxel was used.

Additionally, the NHSA has emphasized that through continuous adjustments to the healthcare security drug list, a majority of commonly utilized clinical antitumor drugs have been incorporated into the scope of basic medical insurance payment. This inclusion effectively caters to the fundamental medical requirements of residents. Through drug price negotiations and centralized procurement, prices for numerous antitumor drugs, including ICIs, have experienced a reduction ranging from 30 to 70%. Consequently, with these negotiated price reductions in place for antitumor drugs, the burden on cancer patients will be further alleviated.

The present study also possessed certain limitations. Frist, considering the differences in cost inputs and payment threshold in different countries, the results of this study may not be applicable to other countries. Second, due to the lack of head-to-head comparative studies on PD-1/PD-L1 inhibitors, particularly in terms of domestic versus imported agents, which undermines the persuasiveness of this study. Third, the studies we included exhibit certain limitations, such as inconsistent follow-up durations across different countries. Thus, further investigation between different PD-1/PD-L1 inhibitors in larger cohorts is needed.

## Conclusion

The administration of tislelizumab in patients with advanced or metastatic NSCLC not only extends the progression-free survival (PFS) and overall survival (OS). Moreover, this treatment exhibits a favorable cost-effectiveness profile throughout the Chinese population and holds significant implications for China’s healthcare system and clinical practice.

## Data availability statement

The original contributions presented in the study are included in the article/Supplementary material, further inquiries can be directed to the corresponding author.

## Author contributions

XZ: Conceptualization, Data curation, Formal analysis, Writing – original draft, Funding acquisition, Investigation, Methodology, Project administration, Resources, Software, Supervision, Validation, Visualization. XF: Data curation, Formal analysis, Investigation, Methodology, Software, Writing – original draft. JZ: Data curation, Writing – review & editing. FJ: Supervision, Writing – review & editing. YW: Data curation, Writing – review & editing. BY: Software, Writing – review & editing. XL: Data curation, Writing – review & editing. DL: Conceptualization, Project administration, Resources, Supervision, Writing – review & editing.

## Footnotes

^1^https://automeris.io/WebPlotDigitizer/

^2^https://www.yaozh.com/
