# Supplementary material for: Cost-effectiveness analysis of the tislelizumab versus docetaxel for advanced or metastatic non-small-cell lung cancer in China
Source: Front Public Health. 2024 Jul 18;12:1425734. doi: 10.3389/fpubh.2024.1425734 (PMC11291238; doi:10.3389/fpubh.2024.1425734)
Supplement: Supplementary file 1 [file Data_Sheet_1.docx]

**Cost-effectiveness analysis of the tislelizumab versus docetaxel for advanced or metastatic non-small-cell lung cancer in China**

Xiaoyu Zhang^1, *^, Xiongxiong Fan^1, *^, Jin Zhang^1,2^, Fengli Jiang^1,2^, Yiping Wu^1^, Beibei Yang^1^, Xinghuan Li^1^, Dong Liu^1,2^

^1^From Clinical Pharmacy Office, Baoji Central Hospital, Baoji, Shaanxi, China

^2^Department of Pharmacy, Xi’an Jiaotong University Health Science Center, Xi’an, Shaanxi, China

*These authors contributed equally to this work.

Correspondence to: Dong Liu, Clinical Pharmacy Office, Baoji Central Hospital, Baoji, Shaanxi, China, 8 Jiangtan Road, Weibin District, Baoji City, Shaanxi Province. E-mail: [liudong691122@126.com](mailto:liudong691122@126.com) (D. L.)

**Table S1. Comparison of parameters of K-M curves after reconstruction.**

| **Parameter** | **RATIONALE-303** | **Reconstructed curve** |
| --- | --- | --- |
| HR | 0.63 | 0.6154 |
| L95% | 0.53 | 0.5194 |
| U95% | 0.75 | 0.7292 |

L95%: 95% lower limit; U95%: 95% upper limit.

**Table S2. AIC and BIC statistics for alternate parametric distributions.**

| **Parameter** | **AIC** | | **BIC** | |
| --- | --- | --- | --- | --- |
|  | **Tislelizumab** | **Docetaxel** | **Tislelizumab** | **Docetaxel** |
| OS |  |  |  |  |
| Weibull (AFT) | 3017.35 | 1536.78 | 3025.92 | 1543.97 |
| log−Normal | 3007.19 | 1522.88 | 3015.76 | 1530.07 |
| log−Logistic | 3001.65 | 1524.55 | 3010.21 | 1531.75 |
| Gompertz | 3025.62 | 1541.66 | 3034.18 | 1548.85 |
| Gen. Gamma | 3005.65 | 1524.73 | 3018.50 | 1535.53 |
| Gamma | 3013.75 | 1533.83 | 3022.32 | 1541.02 |
| Exponential | 3023.88 | 1539.70 | 3028.16 | 1543.30 |
| PFS |  |  |  |  |
| Weibull (AFT) | 2896.63 | 1067.67 | 2905.19 | 1074.86 |
| log−Normal | 2785.19 | 1008.19 | 2793.75 | 1015.38 |
| log−Logistic | 2794.00 | 999.89 | 2802.57 | 1007.09 |
| Gompertz | 2838.56 | 1074.13 | 2847.13 | 1081.33 |
| Gen. Gamma | 2759.20 | 1008.55 | 2772.05 | 1019.34 |
| Gamma | 2907.09 | 1052.76 | 2915.65 | 1059.95 |
| Exponential | 2907.25 | 1075.15 | 2911.53 | 1078.75 |


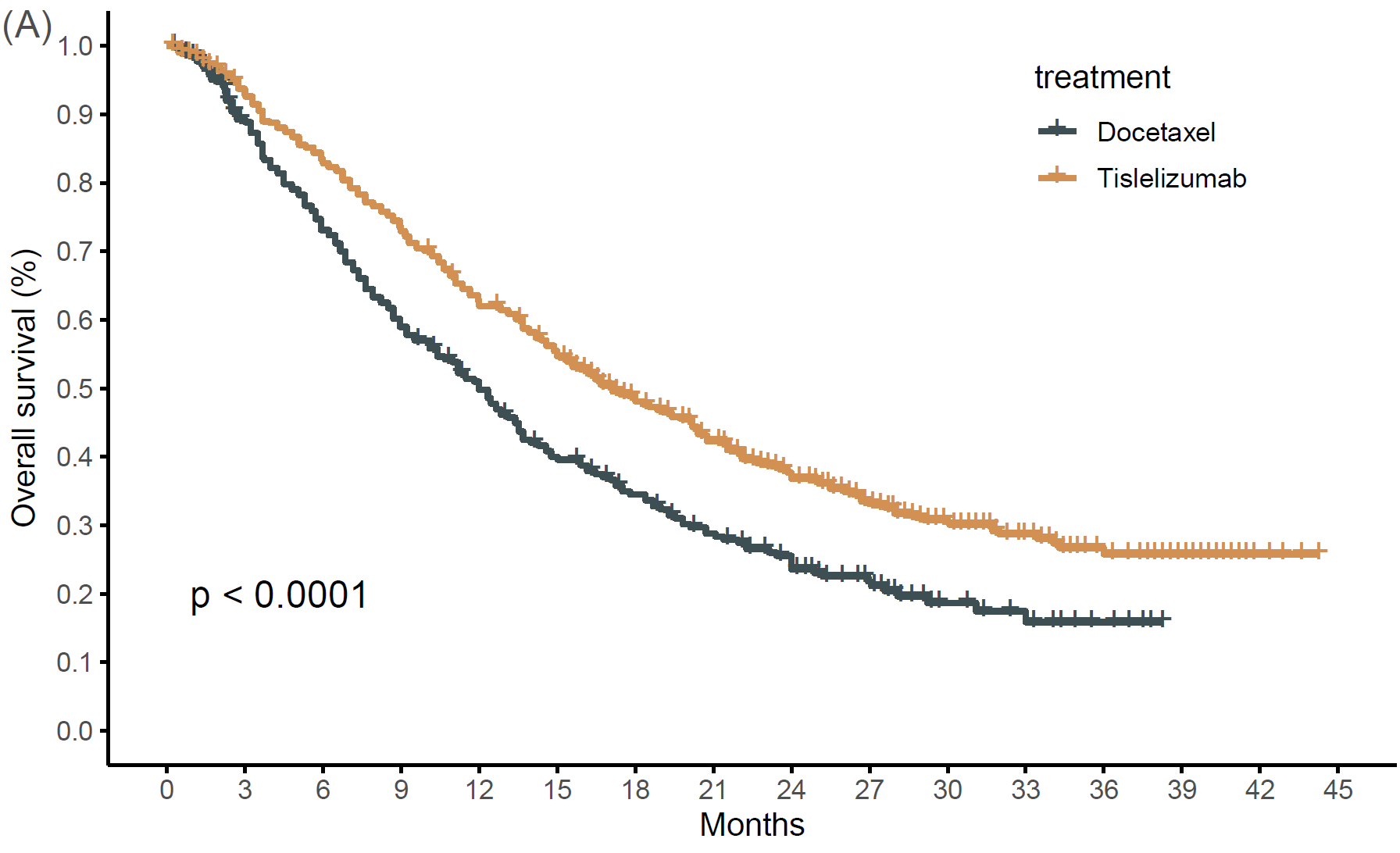


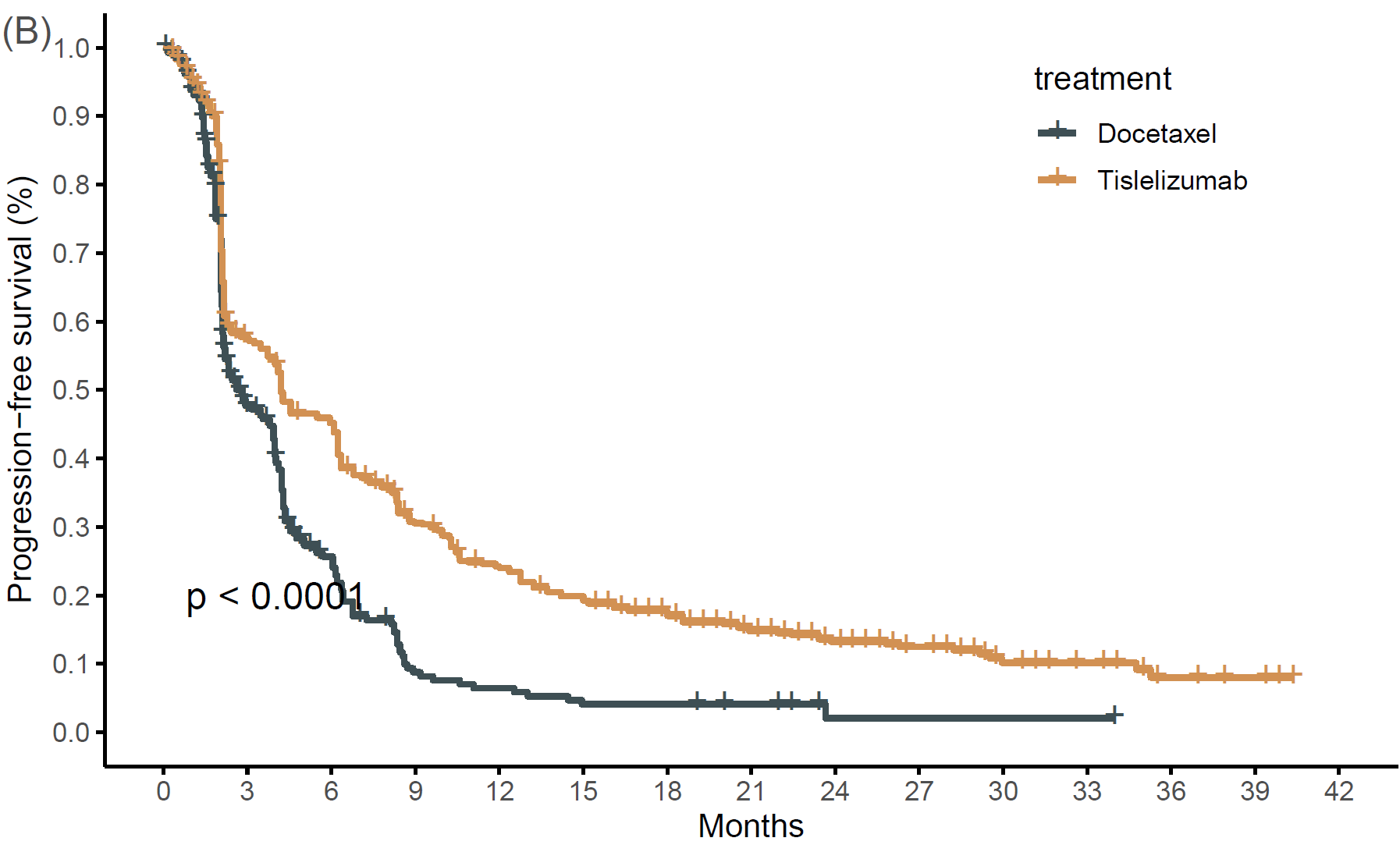


**Figure S1.** (A) Reconstructed OS curve; (B) Reconstructed PFS curve.


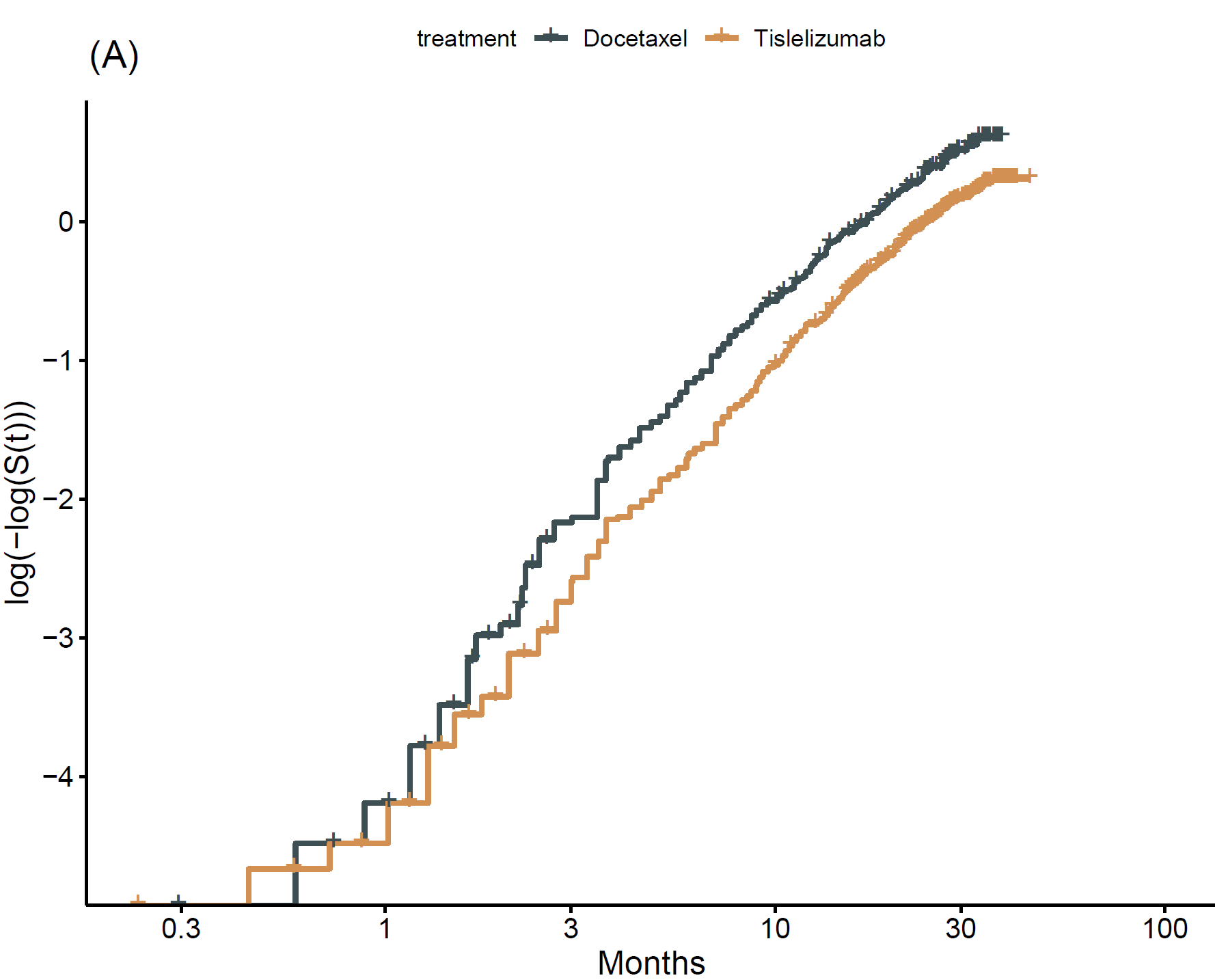

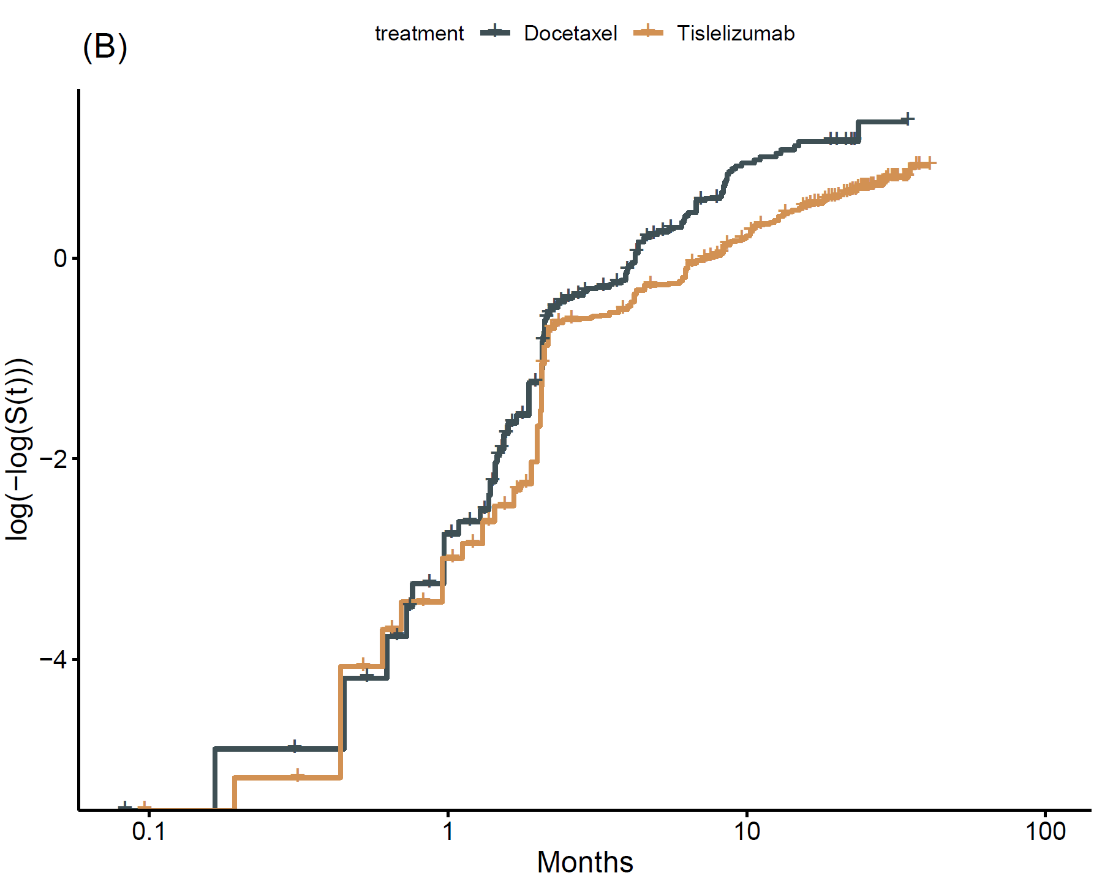


**Figure S2.** (A) Proportional Hazard Model of OS data; (B) Proportional Hazard Model of PFS data.
